# Descemet Membrane Endothelial Keratoplasty - Complication and management of a single case for tissue preparation and graft size linked to post-op descemetorhexis disparity

**DOI:** 10.1016/j.ajoc.2018.09.003

**Published:** 2018-09-06

**Authors:** Mohit Parekh, Alessandro Ruzza, Abigail Kaye, Bernhard Steger, Stephen B. Kaye, Vito Romano

**Affiliations:** a UCL Institute of Ophthalmology, London, United Kingdom; b International Center for Ocular Physiopathology, The Veneto Eye Bank Foundation, Venice, Italy; c Department of Ophthalmology, St. Paul's Eye Unit, Royal Liverpool University Hospital, Liverpool, United Kingdom; d Department of Ophthalmology, Medical University of Innsbruck, Innsbruck, Austria; e Department of Eye and Vision Science, University of Liverpool, Liverpool, United Kingdom

**Keywords:** Endothelial keratoplasty, DMEK

## Abstract

**Purpose:**

To report the management of an intraoperative complication during large (9.5 mm) ultra-thin Descemet Stripping Automated Endothelial Keratoplasty (UT-DSAEK) surgery in a patient with a large area of dysfunctional endothelium.

**Observations:**

A single case study of an 89 y/o male with a history of Fuchs corneal endothelial dystrophy is presented. The patient was listed for a large UT-DSAEK, but due to an intraoperative complication during graft preparation, an 8.00 mm Descemet membrane endothelial keratoplasty (DMEK) was prepared from the same graft using a standardized SCUBA technique and delivered. Early postoperative examination of the graft showed decentred, residual corneal oedema in the absence of DM detachment and a well-formed anterior chamber. The endothelial graft was found attached after 3 months and the corneal oedema was cleared. After 5 months, the patient's BSCVA was recorded at 6/6(20/20) in the left eye, but complained of mild discomfort. A circular ring of corneal oedema was observed around the graft and decentralization of the transplanted graft was observed. Endothelial cell density (ECD) of the central cornea at 5th month was 1506 cells/mm^2^ at a focal depth of 496 μm with some polymegathism.

**Conclusions:**

*and importance*: It is possible to prepare DMEK starting from a failed DSAEK graft. Thickness map on corneal tomography could be a useful tool after DMEK for checking graft centration, function, and corneal recovery indirectly. It is recommended to only maintain a small distance between the descemetorhexis area and the size of the endothelial graft.

## 1 Introduction

Endothelial Keratoplasty (EK), especially Descemet Stripping Automated Endothelial Keratoplasty (DSAEK) has become a gold standard for keratoplasty procedures.^1^ However, with advancements seen since the last decade, Descemet Membrane Endothelial Keratoplasty (DMEK) has also been a front-runner for treating EK cases, with advantages that include better visual recovery and early rehabilitation rates compared to penetrating keratoplasty (PK).^2^, ^3^, ^4^ DSAEK graft preparation methods are already standardized with the use of a microkeratome. Whereas DMEK is still being standardized due to the technical challenges that are observed during graft preparation and implantation. A DMEK graft is usually prepared from a PK tissue.

We report a case where the patient was deemed suitable for Ultra Thin (UT-DSAEK) but due to the preparation of a non-uniform graft after microkeratome cutting with poor stromal bed, the tissue was further stripped from an UT-DSAEK graft and transplanted as a DMEK graft. We have also noted that matching the size of descemetorhexis with the graft size is important in order to avoid peripheral stromal edema and reduce the detachment rate. Thus, the aim of this single case report is to show that a DMEK graft can be prepared even after a failed DSAEK preparation further reducing possible wastage of tissue.

## 2 Case report

**Fig. 1:**
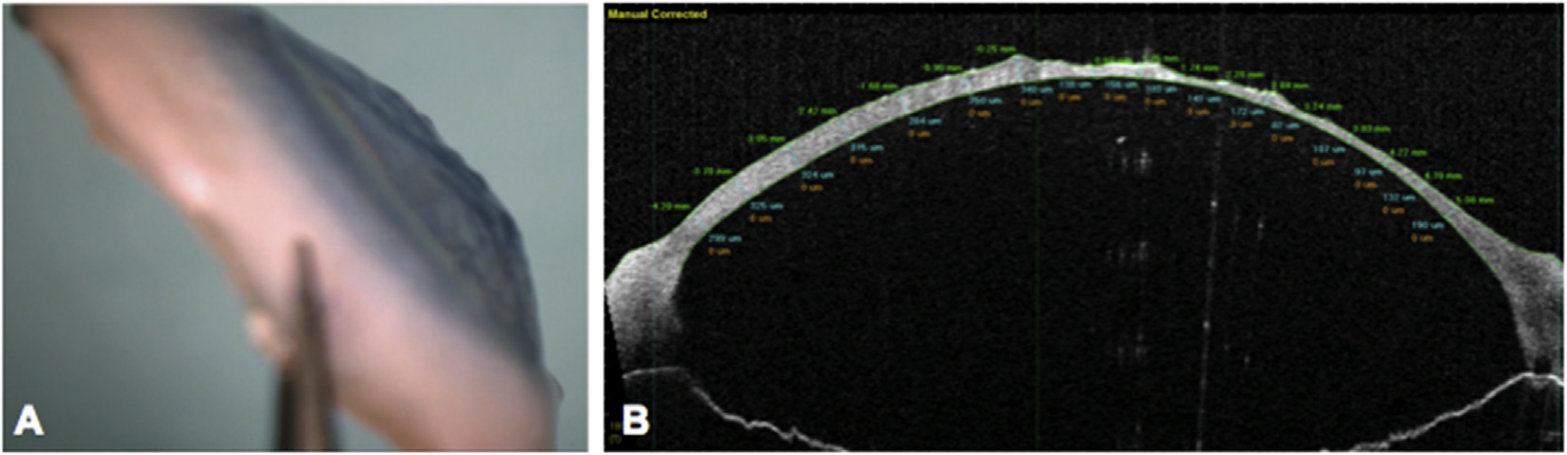
A) Donor cornea and B) OCT image - showing irregular stromal surface due to loss of vacuum during UT-DSAEK preparation.

**Fig. 2:**
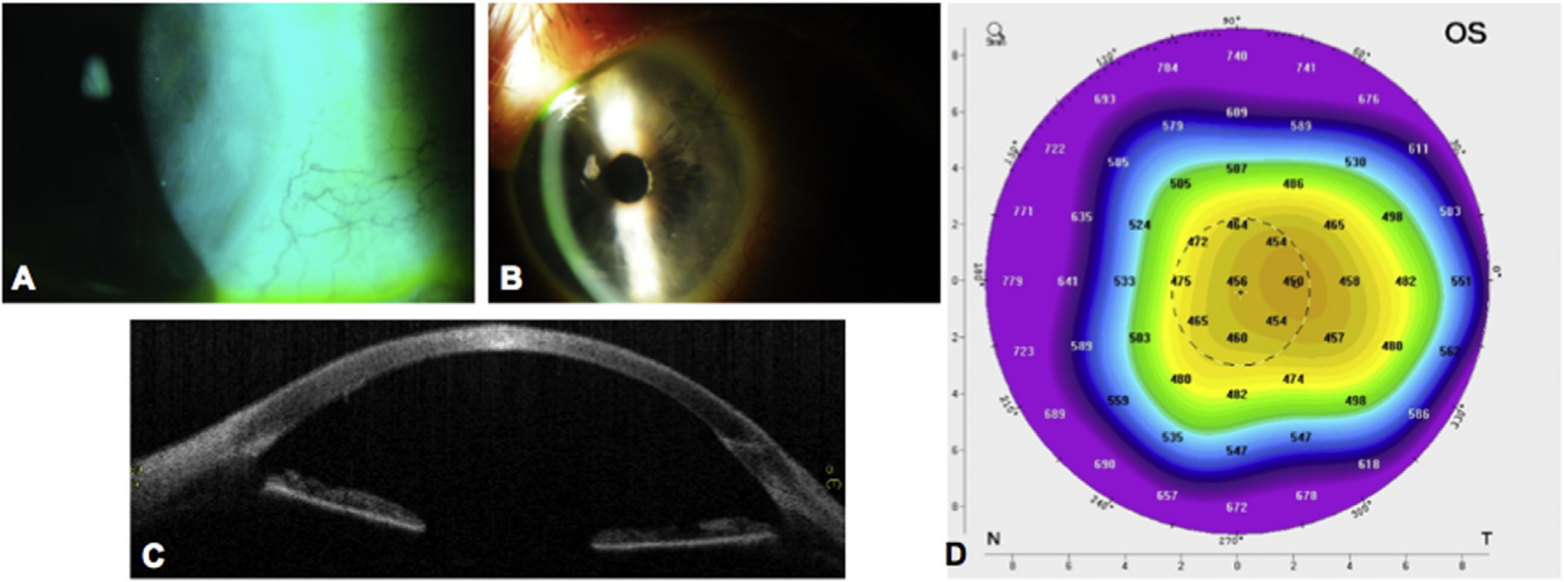
A and B) Left eye of the patient showing circular corneal oedema all around the corneal graft, C) OCT image of the cornea with decentered DMEK graft and D) Thickness map showing decentered DMEK graft and increased corneal thickness of the exposed or bare stroma without the DMEK graft or endothelial cells compared to the central region.

An 89-year-old man with a history of Fuchs corneal endothelial dystrophy presented in the left eye with reduced visual acuity and discomfort due to the development of pseudophakic corneal edema following previous uneventful cataract surgery in 2005. The best spectacle corrected distance visual acuity (BSCVA) was 6/18 (20/63) in the right eye and 6/24(20/80) in the left eye. Intraocular pressure was 12 mmHg in both the eyes with central corneal thickness in the left eye of 658 microns, measured using ultrasound pachymeter. The guttae and resulting endothelial dysfunction involved most of the endothelial surface, therefore, a large (9.5 mm) ultra-thin Descemet stripping automated endothelial keratoplasty (UT-DSAEK) was planned. The graft was prepared following our previously published protocol.^5^^,^^6^ However, due to loss of vacuum on the Barron trephine during cutting resulted in an irregular stromal surface (Fig. 1A), which was verified using anterior segment optical coherence tomography (AS-OCT) (SS-1000 Casia; Tomey Corporation, Nagoya, Japan) (Fig. 1B). In order to reduce the corneal wastage, a Descemet membrane endothelial keratoplasty (DMEK) was performed in his left eye using an 8.00 mm donor graft (prepared using SCUBA technique, video 1) placed within a recipient descemetorhexis of about 9.5 mm. Delivery and unfolding of the tissue was achieved without intraoperative complications. Topical prednisolone acetate 1% (Pred Forte, Allergan) and chloramphenicol 0.5% eye drops (chloramphenicol) four times a day were used postoperatively. An early postoperative examination of the graft showed it to be slightly temporally decentred, residual corneal edema in the absence of DM detachment and a well-formed anterior chamber. However, the endothelial graft remained attached and the corneal edema had cleared. After 3 months, AS-OCT (SS-1000 Casia; Tomey Corporation, Nagoya, Japan) confirmed that the graft was completely attached. After 5 months, the patient's BSCVA was recorded at 6/6(20/20) in the left eye, but with complain of mild discomfort. His left cornea was clear in the centre, but it showed a circular ring of corneal edema around the graft (Fig. 2A and B) evident on corneal tomography (Pentacam, Oculus, Germany). Corneal map (Fig. 2C) and AS-OCT (Fig. 2D) for thickness measurement showed decentralization of the transplanted graft with edema observed in the corneal map (Fig. 2C). Endothelial cell density (ECD) of the central cornea assessed using *in vivo* confocal microscopy (IVCM, Heidelberg Retina Tomographer II with Rostock Cornea Module; Heidelberg Engineering, Heidelberg, Germany) 5 months after surgery was 1506 cells/mm^2^ at a focal depth of 496 μm with some polymegathism.

Supplementary video related to this article can be found at https://doi.org/10.1016/j.ajoc.2018.09.003.

The following is the supplementary data related to this article:

Video 1 Descemet Membrane Endothelial Keratoplasty (DMEK) graft preparation from a failed Descemet Stripping Automated Endothelial Keratoplasty graft. The video illustrates the method of stripping a DMEK graft from an irregularly cut Ultra Thin-DSAEK graft due to loss of vacuum while cutting UT-DSAEK in the surgical theatre.1 Video 1

## 3 Discussion

This case report highlights some interesting points. It is possible to prepare a DMEK lenticule starting from a posterior lamellar graft (DSAEK). In cases of irregular donor surfaces it is still a valid and achievable option. A multimodal approach AS-OCT and the thickness map on corneal tomography are useful tools after DMEK for indirectly checking graft centration, function, attachment, and corneal recovery. Melles et al. have previously reported the utility of corneal tomography in the follow-up to highlight early signs of rejection after DMEK.^7^ The size of descemetorhexis in DMEK should be slightly smaller compared to the diameter of the graft. It should be noted, that graft diameter or chord length in the eye differs from the diameter of the graft on the trephine block.^8^ This difference depends on the disparity between the radius of curvature of the trephine and that of the posterior cornea. The radius of curvature can be 9.5 mm (Hessburg-Barron trephine block), 7.5 mm (Moria trephine block) or 7.7 mm (Janach trephine block). The circumferential arc length of the trephined graft is the product of the trephine size (Y) and angle (radians). For example, for a trephine size of 8.0 mm on a trephine base of radius R_1_ of 9.5 mm, the circumferential arc length is 9.5mm × α_1_, where α_1_ can be calculated from the function, α_1_ = 2Arcsin(Y_1_/2R_1_). Therefore, for an 8.00 mm sized graft, α_1_ = 2Asin(4.00/2 × 9.5) or 1.05 radians or 60°, giving a circumferential arc length C of 8.25 mm. If the graft assumes a radius of curvature on the posterior corneal surface R_2_ in the eye of 5.70 mm, then α_2_ = (C/R_2_) or 1.75 radians or 100°. The expected chord length (Y_2_) of the graft in the eye is therefore Y_2_ = 2 (5.7) sin 50° or 7.75 mm. Therefore, for a 8 mm donor DMEK preparation we will have a smaller graft size of 7.75 mm in the eye using Barron, 7.9 mm using Moria, 7.88 mm using Janach. There is always an under sizing of the graft compared to the trephine used. This under sizing increases with larger diameter graft. Migration of endothelial cells to cover the gap between the edge of the graft and host Descemet's membrane and endothelium may not be a consistent event and in this case by 5 months, appeared insufficient to have bridged the circumferential gap of 1.75mm (9.5mm - 7.75mm).^9^ On the basis of better graft survival following larger grafts in DSAEK and knowing that the technical difficulties of manipulating a larger graft can be overcome,^10^ we would suggest using larger endothelial grafts with a donor trephine size of 9 mm or 9.5 mm.^8^ Since there are many factors that contribute to endothelial graft failure, the health of the donor endothelium is likely to be pivotal. It is reasonable to suggest, therefore, that transplanting larger grafts and replacing a larger proportion of diseased endothelial cells may lead to improved graft survival. Although this is just a single case observation, it reveals that it is possible to prepare a DMEK graft from an UT-DSAEK donor and that there may be a certain limitation of the donor endothelial cells to recover the exposed stromal area, it would however be beneficial to increase the size of the graft if the endothelial dysfunction affects a large area. The gap between a DMEK graft and the recipient periphery should be minimal to reduce the risk of peripheral corneal oedema.

## Patient consent statement

The corneas were harvested by Fondazione Banca degli Occhi del Veneto Onlus for transplantation purpose after obtaining a written consent from the donor's next-of-kin.

A written consent from the patient was obtained before transplantation.

## Funding statement

None of the authors have any financial interest.

## Conflicts of interest

None of the authors have any conflict of interest.

## Authorship statement

All authors attest that they meet the current ICMJE criteria for authorship.

MP, AR, SK, VR - Substantial contributions to the conception or design of the work; or the acquisition, analysis, or interpretation of data for the work; AND.

MP, AK, BS, VR - Drafting the work or revising it critically for important intellectual content; AND.

MP, AR, AK, BS, SK, VR - Final approval of the version to be published; AND.

MP, AR, AK, BS, SK, VR - Agreement to be accountable for all aspects of the work in ensuring that questions related to the accuracy or integrity of any part of the work are appropriately investigated and resolved.
